# The impact of chemotherapy-induced inner ear damage on quality of life in cancer survivors: a qualitative study

**DOI:** 10.1007/s11764-021-01089-5

**Published:** 2021-08-16

**Authors:** Stephanie E. Pearson, Charlotte Caimino, Maryam Shabbir, David M. Baguley

**Affiliations:** 1 Nottingham Biomedical Research Centre, National Institute for Health Research, Ropewalk House, 113 The Ropewalk, Nottingham, NG1 5DU UK; 2 Hearing Sciences, Mental Health and Clinical Neurosciences, School of Medicine, University of Nottingham, University Park, NG7 2RD UK; 3 Nottingham University Hospitals NHS Trust, Nottingham, UK

**Keywords:** Survivorship, Quality of life, Hearing loss, Tinnitus, Late effects

## Abstract

**Purpose:**

This study aimed to explore the burden of inner ear damage (ototoxicity) on adults living with and beyond cancer treated with chemotherapy and  the impact on their quality of life (QoL). Furthermore, this study aimed to explore patient awareness surrounding chemotherapy-induced inner ear damage, known as ototoxicity, and assess what support they had been offered.

**Methods:**

Participants were adults who had undergone chemotherapy, recruited from cancer clinics, charities and social media. Using semi-structured interviews and fieldnotes, an inductive thematic analysis was used to develop key themes surrounding this topic.

**Results:**

Twenty participants from the UK were interviewed. Two key themes were developed from the thematic analysis, cancer-related QoL and ototoxicity-related QoL, with each one including 5 subthemes. Subthemes consisted of impact of ototoxicity, hearing, tinnitus, clinical experience, audiological assessments, and impact of treatment, cancer and chemotherapy, other toxicities, information and patient reflections.

**Conclusions:**

Ototoxicity can have a negative impact on QoL, specifically on social life and the fear of hearing loss and/or tinnitus worsening. There are opportunities for increased awareness by patients and clinicians, including improved information sources, and hearing monitoring not only for those undergoing platinum-based chemotherapy but many others surviving after treatment for cancer.

**Implications for Cancer Survivors:**

Better monitoring of hearing and information about ototoxicity during chemotherapy could potentially reduce the fear of the symptoms of ototoxicity worsening. Furthermore, hearing monitoring would facilitate the detection of hearing loss at early stages of survivorship, which would facilitate earlier access to clinical interventions and longer term counselling.

## Introduction

While the overall 5-year survival rate for adult cancer is above 80%, many adults living with and beyond cancer (LWBC) face long-term, often permanent, physical and psychological hardships from treatment [1–3]. These long-term or late-effects, such as peripheral neuropathy, can occur months after treatment and can severely impact quality of life (QoL) [4, 5]. QoL has become increasingly important in health care, as outcome measures have progressed beyond biological functioning and morbidity [6]. Although there are multiple definitions of QoL, the World Health Organization (WHO) defines QoL as an “individual’s perception of their position in life in the context of the culture and value systems in which they live and in relation to their goals, expectations, standards and concerns. It is a broad ranging concept affected in a complex way by the person’s physical health, psychological state, personal beliefs, social relationships and their relationship to salient features of their environment” [7–10].

There is a substantial literature considering how cancer can impact different aspects of QoL, for example, experiences from ethnic minorities, experiences of returning to work and the impact cancer has on sexuality [11–15]. Yet, there is a scarcity of research on experiences of ongoing symptoms, such as late effects and the impact they have on QoL [16].

Although platinum-based chemotherapy such as cisplatin, while highly effective, is known to cause ototoxicity [17]. Ototoxicity is defined as drug-induced damage to the inner ear caused by an ototoxic drug, presenting as high frequency hearing loss and tinnitus which can be progressive and irreversible [18–20]. Both hearing loss and tinnitus are associated with a higher risk of developing various comorbidities [21]. Hearing loss is associated with depression, social isolation and dementia [21–24]. Tinnitus is associated with insomnia, poor concentration and anxiety [25–27]. The quality of social interactions for a person with hearing loss and/or tinnitus is often reduced, as taking part in conversations becomes challenging [28].

The impact of hearing loss and tinnitus on QoL in people LWBC remains unclear and under-studied. Cancer treatments can potentially cause life-threatening side effects such as cardiotoxicity and nephrotoxicity, therefore these tend to become the priority [29, 30]. However, once the acute side effects subside or managed, other long-term side effects, such as ototoxicity, remain and can potentially reduce QoL [31]. Cancer survivors may have already experienced a challenging journey from the diagnosis itself, the physical challenges of treatment and finally, remission. Re-adapting back to their previous life while in remission, but with added permanent late effects, both physically and psychologically, can be extremely difficult for some survivors [32].

Currently, there is little information and support offered to patients who suffer from ototoxicity, potentially leading to many being undiagnosed and untreated [33]. As ototoxicity is often permanent, without the appropriate support and guidance to manage these symptoms, there could be a detrimental impact on QoL [32]. It is essential that a deeper understanding and increased awareness of how hearing loss and tinnitus specifically affect the QoL of cancer survivors is sought. Thus, a tailored and personalised support system can be developed to improve management of long-term symptoms.

This qualitative study aimed to explore, in depth, the burden of hearing loss and tinnitus on adult cancer survivors who had been treated with chemotherapy 6 + months prior. Using semi-structured interviews, the specific impact ototoxicity has on QoL was investigated. Furthermore, this study aimed to explore patient awareness surrounding ototoxicity and assess what support had been offered during their treatment and after-care.

## Methods

### Sample

This study is part of a larger mixed-methodology study in which the severity of ototoxicity and the impact it has on QoL was measured using questionnaires, interviews and high-frequency hearing tests. Participants were recruited from National Health Service (NHS) (Nottingham University Hospital NHS Trust and Sherwood Forest Trust) and non-NHS sites including: the Ear Foundation, MacMillan Information Centres at Kings Mill Hospital, Nottingham City Hospital and Queens Medical Centre, Late Effects Clinic at City Hospital, Nottingham Biomedical Research Centre (NIHR BRC) Database, Maggie’s Centre, British Tinnitus Association (BTA) website, Facebook and Twitter and at the Oncology Germ Cell Follow-up clinics. Many more community groups were contacted during this process. Furthermore, a press release was written by the University of Nottingham media team, alongside a patient and public involvement (PPI) representative. The process for obtaining participant informed consent was in accordance with the research ethics committee (REC) guidance, and Good Clinical Practice (GCP). All participants provided written informed consent.

Potentially eligible participants once identified, were screened against the inclusion and exclusion criteria. Inclusion criteria consisted of any adult (≥ 18 years) experiencing self-reported or diagnosed hearing loss and/or tinnitus 6 + months following primary chemotherapy treatment. Exclusion criteria consisted of any person who received radiotherapy to the head and neck area. Radiotherapy to the head and neck area was excluded due to being a confounding issue. Radiotherapy to the head and neck area typically impacts hearing due to radiotherapy being a localised treatment, thus it would be difficult to assess whether it is the platinum-based chemotherapy or the radiotherapy to the head and neck inflicting ototoxic damage.

### Data collection

The initial aim for recruitment was 30 participants; however, data saturation was reached at 20 participants. Data saturation is defined as the point at which data collection provides no novel information [34].

The interviews were carried out in person and audio-recorded by the researcher in a quiet room or setting; however, field notes were also taken as a precaution in case of poor-quality audio. The interviewer was a female PhD student who had no prior relationship to the participants, and a female audiologist was present during the interviews to take field notes. Interviews were semi-structured in-depth interviews, which are the gold standard method of qualitative data collection aiming to investigate illness-related experiences [35].

### Data analysis

Interviews were transcribed verbatim, and all file names were coded. Any identifiable data were removed in the transcription process for anonymity. Furthermore, all files were password protected, encrypted and stored on a University of Nottingham laptop using a secure network.

Interviews were thematically analysed using the Braun and Clarke [36] methodology, with an inductive approach. First, interviews were familiarised with by reading and re-reading the transcripts. Codes were created by highlighting and making notes on key findings using NVivo v12, a qualitative analyses software. Codes were then refined, by a process of condensing, merging and adding to the initial codes. Finally, once the codes were refined, a coding manual was created and sent to the second coder. Discrepancies were resolved to improve the clarity and descriptions of the codes, and finally, these were grouped into themes. The themes were discussed, and a final version of the coding manual was developed to reflect the shared experiences and understandings of the participants.

## Results

From the 20 participants, 8 were female, and 12 were male. Ages ranged between 25 and 77 (mean 53.1, SD 15.3 years). All participants were either White British (18), White European (1) or White Australian (1).

Basic histories were taken from the participants, including type of cancer, type of chemotherapy undergone and any pre-existing auditory issues (Tables 1, 2 and 3).

**Table 1:** Displaying the demographic characteristics from the 20 participants in this study

Demographics		
Age	Mean	53.1
	Range	25–77
	SD	15.3
		N
Gender	Female	8
	Male	12
Relationship status	Single	5
	Living with partner	1
	Married	12
	Widowed	2
Education level	Comprehensive school (e.g. GCSEs)	8
	Further education (e.g. A-levels)	5
	Higher education (e.g. University)	4
	Postgraduate education (e.g. PhD)	3
Employment status	Student	1
	Unemployed	1
	Employed	10
	Retired	6
	Sick leave	2

**Table 2:** Displays the medical characteristics from the 20 participants in this study

Medical participant characteristics		
Years since chemotherapy	Median	4.5
	Range	0.5–20
	Interquartile range	2–6.75
		N
Type of chemotherapy	Cisplatin	7
	Carboplatin	3
	Oxaliplatin	1
	Unknown	5
	Other	4
Site of cancer	Stomach	1
	Breast	5
	Testicular	7
	Multiple myeloma	2
	Bowel	2
	Acute myeloid leukaemia	2
	Cervical	1
Pre-existing auditory status	None	12
	Grommets	1
	Hearing impaired	2
	Tinnitus	4
	Increased sensitivity to sound	1

**Table 3:** Displays examples of quotations from participants which aided the development of themes and subthemes

Theme	Subtheme	Quote
1. Ototoxicity Related Quality of Life	**1.1 Audiological Assessment**	*No, no I didn’t have any [hearing] baseline, but for other things I did” (P19)*
	**1.2 Clinical Experience**	*“She [oncologist] talked me through everything, and everything was clear and weighted.” (P13)*
	**1.3 Hearing**	*“I just couldn’t hear, especially if people spoke softly, or women and children’s voices. I just couldn’t hear them.” (P1)*
		*“It would have been about halfway through my main block of treatments, after the third week maybe? By the fifth week I noticed a definite loss, but I can’t remember when it started exactly.” (P18)*
	**1.4 Tinnitus**	*“It’s certainly a high-level hiss now, in both ears. It’s constant. It never goes away.” (P14)*
		*“It [tinnitus] does as it pleases. I try my best to ignore it and that seems to work.” (P4)*
	**1.5 Impact of Ototoxicity**	*“I’m knackered and it’s just hiss. People can stand in front of me and speak and I’m stressing because I just hear hiss.” (P14)*
		*“I struggled to engage in anything because the tinnitus was frustrating, I wouldn’t engage in much more.” (P16)*
		*“For my sleep I take sedatives. I hear it [tinnitus] when it’s quiet but I deal with it because I know at some point, I’ll be asleep, and it’ll be gone.” (P6)*
		*“It is what it is, it’s an inconvenience, but I’m alive.” (P10)*
2. Cancer Related Quality of Life	**2.1 Cancer and Chemotherapy**	*“The only time I saw my chemo was when it was in a bag, that’s all I know.” (P8)*
		*“I had intravenous treatment for two hours, two weeks of tablets then a week off. I had a lot of cycles.” (P9)*
	**2.2 Other Toxicities**	*“I find I lose my balance sometimes, sort of as if you’re walking and you’re standing on a plank.” (P1)*
		*“After the chemotherapy I felt I had sponges underneath my feet. I have to physically lift my legs up because my feet stick to the floor, I have to be careful with them because I trip up.” (P9)*
	**2.3 Impact of Treatment**	*“After I finished treatment, I left the hospital, that’s when the biggest side effects really hit me.” (P18)*
		*“It was mainly my family and friends that supported me. I was too busy during treatment to go there [charities/information centres].” (P12)*
	**2.4 Information**	*“When you’re in hospital everything is overwhelming, later on when you’re out of hospital and in follow up everything sinks in a bit more.” (P16)*
		*“There was a long list of side effects indeed, but in some sense they were weighed. The doctor went through them all with me and explained which ones were more common and so on. Tinnitus was mentioned I remember that very clearly.” (P17)*
	**2.5 Patient Reflections**	*“It would have been nice to just be a bit more aware of the long-term effects, to prepare myself a bit more for them.” (P10)*
		*“It’s [cancer] not nice, but it’s doable. It’s really one hell of a journey, but it’s doable.” (P14)*

**Fig. 1:**
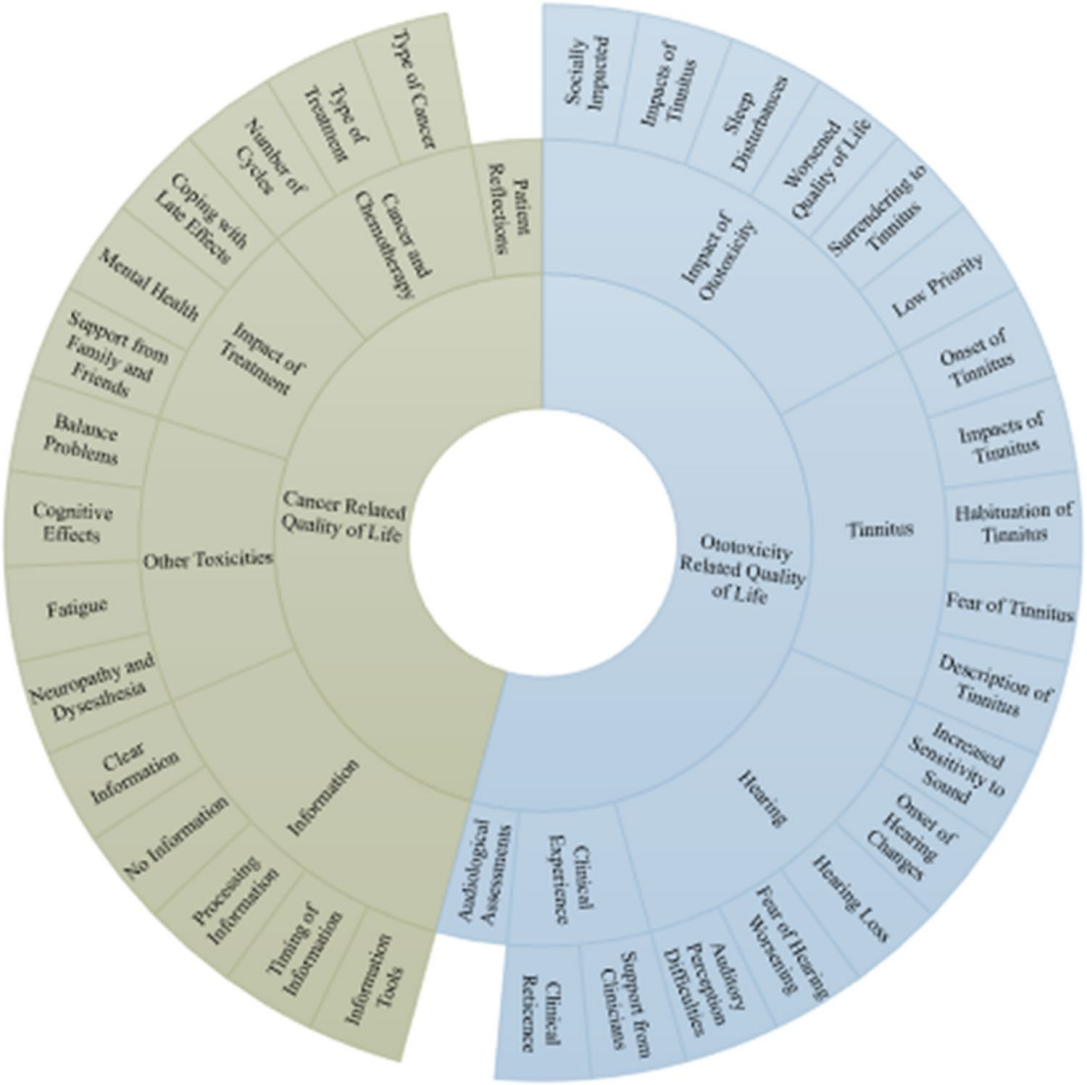
Is a sundial displaying the main themes, and subthemes. The outer ring displays the codes used in the analysis which were grouped to develop the themes and subthemes. Theme 1: Ototoxicity related quality of life and Theme 2: Cancer related quality of life

Using the bottom-up (inductive) thematic analysis methodology, 34 codes were developed from the interview transcripts and fieldnotes. Codes were grouped into themes and subthemes via discussion with a second researcher. During this process it was clear that these could be categorised into those which were ototoxic specific and those which were more generally related to cancer and cancer chemotherapy. Thus, two main themes emerged from the data: *Ototoxicity related quality of life* and *Cancer related quality of life* with five subthemes each. The themes and subthemes can be seen in Fig. 1.

### Theme 1: Ototoxicity related quality of life

The theme ‘ototoxicity related quality of life’ was developed as a result of the experiences described by participants regarding how their ototoxic symptoms (hearing loss and tinnitus) specifically are impacting their quality of life. During the interview process the participants spoke about how ototoxicity directly and indirectly impacts their daily living. They often reflected on the audiological assessments, or lack of, they underwent during their chemotherapy, and they often described experiences with their clinicians when discussing any ototoxic symptoms they had developed. These are explored more in-depth below.

#### Audiological assessments

During the interviews, participants were asked if they had ever had, or been offered, a baseline hearing evaluation prior to starting chemotherapy in order for their hearing to be monitored during their chemotherapy treatment. From the 20 participants included in this study, 19 of them had not been offered any baseline hearing tests prior to chemotherapy treatment. It was highlighted from 1 of the 19 participants that they had received non-hearing related baseline evaluations, but that hearing was never considered.

“No, no I didn’t have any [hearing] baseline, but for other things I did, so I was on Herceptin for a while and before that, I had a baseline echocardiogram. And then after I finished, I then had another one partway through my pattern as well but then certainly no hearing test.” (P19)

Only one participant described was being offered a baseline hearing test. However, despite this offer, the participant explained that they declined as they believed nothing could be done to reverse the impact of chemotherapy on hearing so their results would not matter. This demonstrates that hearing, and ototoxicity more generally, are sometimes overlooked in the treatment of the cancer patients in this study. Incorporating baseline hearing evaluations prior to starting to treatment would be greatly beneficial, and patients hearing should be monitored during treatment to assess any ototoxic effects the participants might be experiencing.

#### Clinical experience

A subtheme that became prominent amongst the participants was their experience with clinicians when mentioning ototoxic symptoms. It became clear that participants encountered two contrasting experiences with their clinicians: support and reticence. Encouragingly, many participants described positive experiences and expressed how supported they felt by their clinical team with regards to ototoxic symptoms there were having.

“She [oncologist] talked me through everything, and everything was clear and weighted.” (P13).

However, for the participants who experienced clinician reticence, they described feelings of being unsupported and that they were not taken seriously when describing their symptoms.

“With the tinnitus I got nothing, I got ‘you have tinnitus, off you go’”. (P2).

It is worth noting, that all participants’ expressed gratitude and appreciation to their care team despite the reticence or support received. Participants were clear in how much they valued their care team and held them in high regard for treating their cancer. However, participants recount discussions of ototoxicity in a much more positive light and describe much more understanding of what their circumstances were when met with support from their care team. The experiences participants highlighted demonstrate that there is great value in having a reassuring and understanding care team, in all aspects of chemotherapy side-effects.

#### Hearing

The hearing subtheme encapsulated both the physiological descriptions of hearing changes the participants mentioned, and the psychological issues surrounding this change in hearing. Many participants described changes to their hearing levels after receiving chemotherapy, which was predominantly a reduction of their ability to hear quieter sounds. The most common hearing issue that was mentioned was auditory perception difficulties. Participants described occurrences of situational or directional hearing loss, such as difficulty hearing in background noise, which is commonly seen in patients who have undergone chemotherapy.

“It’s like there’s gaps. If I turn my head over, suddenly bits of voice would just drop out. It was really directional, so I was constantly having to turn my head to hear what people were saying to me.” (P18)

An unexpected code emerged from the interview transcripts, where participants described having an increased sensitivity to sound. Certain sounds produced new responses in participants which they had not experienced before. These responses resemble similar responses seen in patients who have the hearing disorders misophonia and/or hyperacusis. Misophonia is a decreased tolerance to specific sounds [37] and hyperacusis is the perception of everyday environmental sounds as being overwhelmingly loud or intense [38]. This was also found in a previous study on online health forums, but has not yet been discussed in the medical literature [39].

“A lot of loud noises really started to irritate me quicker than before, like a dog as barking and it just seemed really loud. Once the chemo finished it cleared up but for a few months it was horrid.” (P16)

Participants were asked if they could remember when their hearing deficit began or worsened. The onset of hearing changes ranged from during the first few cycles of chemotherapy, to noticing it a year after chemotherapy ended. The difference in timing of onset of hearing changes further illustrates the importance of monitoring for ototoxic effects not only during treatment, but afterwards. Changes to hearing and tinnitus, like other late effects of chemotherapy, have a critical role in adjusting to life after treatment [32] and should be considered with such importance.

Another critical issue which emerged from the interviews was the fear of hearing worsening. Participants often mentioned feeling fearful of not knowing if their hearing loss will worsen, be permanent, or if there is anything that can be done to prevent further deterioration.

“When it started to deteriorate and go, I thought, I’m going to be totally deaf. Does it come back? Is it going to go up and down? It’s pretty difficult to deal with.” (P18)

Education and information of ototoxicity is therefore key for chemotherapy patients, along with guidance and support on how to deal with any issues should they arise.

#### Tinnitus

Many participants described their experience of tinnitus since having chemotherapy treatment. Participants discussed their individual experiences which included the different types of tinnitus they have such as the frequency and location. There was a combination of both lateral and bilateral tinnitus detailed by participants, as well as the frequency of their tinnitus, with most participants describing a high pitch. Many of the participants discussed having, or attempting, some form of habituation of tinnitus. The importance of habituation in aiding coping and adjusting to tinnitus is longstanding [40], evidence shows that those who habituate to tinnitus have lower levels of tinnitus related distress, anxiety and depression [41]. Some participants explained that they tried to ignore the sound of their tinnitus, while others described a sense of ‘getting used to it’. There were however, mentions of having a fear of tinnitus, where participants would talk about being fearful of their tinnitus worsening.

“I worry I won’t habituate enough, and it’ll get worse. I won’t be able to sleep like I can now.” (P12)

Being fearful of tinnitus has been shown to correlate with not only decreased QoL, but also with having more direct attention towards tinnitus [42] and therefore these patients are less likely to achieve habituation to tinnitus and perceive their symptoms as more severe.

Participants were also asked if they remembered the onset of tinnitus. Four participants had experienced tinnitus prior to undergoing chemotherapy, however for those who had not experienced tinnitus before, the onset varied from the early stages of treatment, to further along in their chemotherapy regime.

“Probably two or three weeks into chemotherapy I started noticing tinnitus in my left ear. It was quite bad for 6 months.” (P16)

Once again, this further underlines the need for continuous monitoring of patients for ototoxicity throughout and after their treatment.

#### Impact of ototoxicity

The impact of ototoxicity on QoL was discussed at length by each participant. There were mentions of feelings of exhaustion from the continuous tinnitus sounds which also caused sleep disturbances, as well as frustration at how their ototoxic symptoms affected their ability to communicate effectively with others. Furthermore, experiences on how ototoxicity socially impacted the participants was commented on. This included hearing loss, tinnitus, or both having an impact on people’s social lives.

“There’s social interactions when you just can’t hear. It’s funny, my ears seem to tune in and out. I can hear certain people a bit better than others. Ladies’ voices- just nothing.” (P14)

The negative effect of decreased social interaction from not only hearing loss and tinnitus, but decreased social interaction in a more general sense, is well known and long established and was evidenced in the interviews with participants. Furthermore, participants spoke about surrendering to tinnitus. This is described as giving in to it in a negative way. Some participants openly spoke about how they feel ototoxicity worsened their QoL in a significant way.

“When you get that diagnosis and you go to the oncologist, they give you chemo. If they had said my hearing was going to go on top of all that, I would have been straight down the pub, my backup retirement plan, which is a large bottle of single malt and a massive pile of paracetamol.” (P14)

However, conversely some participants shared that ototoxicity was a low priority for them, meaning that it did not have a great impact on their QoL compared to other long-term side effects they experience. This outlook was generally seen in participants who viewed ototoxicity as a minor inconvenience in comparison to the effects of cancer prior to chemotherapy.

### Theme 2: Cancer related quality of life

Although the interviews with participants focussed on ototoxicity, some participants expressed how difficult it was to isolate one side effect from another and to identify only one way in how their life has changed since chemotherapy. Although this theme is not specifically related to ototoxicity, it is important to note that these themes are not mutually exclusive. Participants highlighted the impact of treatment, the impact of other toxicities experienced as a result of chemotherapy, more general discussion of cancer and chemotherapy, and finally patient reflections on their cancer journey and experience. These are explored in more detail below.

#### Cancer and chemotherapy

Participants were asked about the type of cancer they had, the type of treatment, and the number of cycles of treatment they received. It was somewhat surprising to hear that many did not know the specific type of treatment they received. Although these developed one subtheme, there were two extreme types of answers and discussion. Some participants were very unaware of the types of chemotherapy treatment they received but were also unperturbed by this lack of knowledge and avoidance of information.

“The only time I saw my chemo was when it was in a bag, that’s all I know.” (P8)

On the contrary, other participants were able to give an in-depth recollection of their treatment which included names of medications, methods of treatment and treatment cycles. This polarity of behaviour of information seeking and avoidance are not uncommon in patients of chronic illness and are employed as coping mechanisms to aid adjustment to illness [43].

#### Other toxicities

Although discussion mainly revolved around ototoxicity, a pattern developed between those experiencing ototoxicity and other toxicities. For example, a few participants mentioned having balance problems. Balance problems are multi-factorial and are associated with neuropathy, weakness or impaired proprioception (perception or awareness of position or movement of the body). Although balance problems can also be associated with vestibular toxicities, the participants felt other comorbidities, such as weakness and neuropathy, were responsible. Neuropathy was mentioned by most participants. This was any mention of chemotherapy-induced peripheral neuropathy (CIPN), and specific mentions of reacting to cool temperatures. A common complaint was having chemotherapy-related cognitive effects, commonly referred to as ‘chemo-brain’.

“I'd lose stuff all the time. I thought I either had a brain tumour because it was it was that bad, and I’m normally such an organised person. I know where everything is, and it wasn't till I went back to the consultant and told him and he said, argh- it’s chemo brain!” (P19)

The term ‘chemo brain’ was a common feature of the discussions with participants and was used frequently by participants. Participants also seemed to use this in conversation with family and friends as a colloquial term and way for them to describe their experiences more casually. Additionally, chemotherapy-related fatigue was also spoken about considerably as a late effect of cancer treatment.

“Although the most difficult part started at the end of last year because I started to develop some side effects that I’m still trying to deal with. In particular, I'm feeling extremely tired.” (P17)

This is perhaps more notable than other effects mentioned in relation to ototoxicity, as there are also recent findings which indicate the effect of hearing impairment of fatigue [44]. Patients who have undergone chemotherapy and are also experiencing a hearing loss could be experiencing fatigue from both things, which in turn exacerbate the other.

#### Impact of treatment

Experiences were discussed by the participants on how their lives were impacted by treatment, both directly and indirectly. Specifically, how the participants’ managed their chemotherapy-related side effects was spoken about frequently in discussion of coping with late effects. It was clear from the discussions that the late effects of the chemotherapy treatment were among the most difficult elements of their experience.

“You have to get on with your normal life as much as you can. However, the treatment just broke me.” (P2)

Another development was the impact this had on mental health. Specifically, how living with, or living beyond cancer impacts mental health.

“I think if you let it get you down, you can let it, but I'm not going to let it get me down. I've been through too much to let this get me down. So I just want to keep doing what I can do from day to day.” (P9)

Finally, support from family and friends and the gratitude the participants felt towards their loved ones was a topic mentioned very frequently.

#### Information

The quality of information that the participants received regarding the potential ototoxic side-effects of their chemotherapy treatment was spoken about in the interviews. Participants described a range of experiences in relation to receiving information about the potential ototoxic side effects. Although some participants did describe being informed, a lot of participants were provided little to no information.

“I don’t recall anything being mentioned about tinnitus at all. I just noticed the tinnitus after the treatment finished when I went home.” (P5)

For those participants who did receive information regarding ototoxicity, discussion included the tools that were used to present the information to participants, which included leaflets and books. It became apparent that the format of the information tools utilised were unsatisfactory to participants. Some described feeling that the use of leaflets as a mechanism to present such crucial information felt valueless as they were unable to absorb this information at this time.

“Yes, I got a lot of leaflets, not really useful to me because I didn’t read them. I was in shock, so I’ve only just gone through and read them years later. I just never looked at them.” (P1)

The timing of information being presented was also a key factor in not only having an awareness of the possibility of ototoxic side-effects, but also an understanding of ototoxicity.

Many felt that processing information was difficult, with some participants expressing difficulties with taking in all the information. All but two participants found the information overwhelming.

“We were just shown too much information and it was overwhelming. It was too much too quickly.” (P1)

#### Patient reflections

A subtheme that emerged from the interviews was patient reflections. This included a wide range of tips, advice, and guidance from the participants on how they made their treatment easier to live with, or how they would help others who are LWBC. This subtheme ranged from positive insights on what services could be implemented or improved, to describing how their mindsets have changed through the cancer journey.

“A baseline audio test would have been helpful, or even a chat with an audio specialist just to sit and chat things through with you, as a standard. Even to warn you, rather than just dealing with it after you get it.” (P18)

## Discussion

The themes derived from the thematic analysis in this study aimed to represent the in-depth experiences of participants and provide insights into the issues surrounding awareness, support, and the impact on QoL from chemotherapy-induced ototoxicity. The findings show that, overall, ototoxicity is not widely known as a side effect of chemotherapy until it is experienced personally, as many participants mentioned not recalling being told about hearing or tinnitus. This may be due to the lack of ototoxicity monitoring being implemented in cancer clinics [45–47], or how participants felt overwhelmed and did not absorb the information when reading leaflets.

Many participants in this study described that they were unaware of the effects of ototoxicity prior to experiencing them. They expressed that the information may have been initially presented to them prior to treatment, but that they were unable to absorb this information due to a feeling of being overwhelmed. This demonstrates that there is a need to ensure that patients are aware of the ototoxic effects of chemotherapy throughout the treatment process and upon treatment completion. Conversations surrounding side effects, including late effects, should be ongoing, at different timepoints during the chemotherapy journey to ensure information can be used effectively. The tools for information delivery also require careful consideration, as many patients described a sense of ‘leaflet overload’. During chemotherapy treatment patients were presented with a large amount of information in leaflet format which they were not able to digest given their current circumstances. Thus, information tools should be used similarly to personalised medicine, where the format and timing of the information, guidance and support should be optimised to the individual. For example, some participants expressed wanting to be warned about the effects early in the treatment process, whereas others felt they would not engage with any information at that time. Further research is needed on how information delivery is most effective, however personalising information to patient needs could be optimised. For example, including audio-visual information such as videos, interactive information such as mobile applications in addition to the traditional leaflets, booklets and books. Furthermore, further research is needed as to when is best to re-engage with this information. It may be helpful for some patients to revisit this information after one cycle, but some may prefer to re-visit later on in their chemotherapy journey to fully acknowledge and understand the various side effects associated with treatment.

From the themes developed, it is suggested that clinical perspectives on tinnitus can be a factor in their patients’ QoL outcomes. It is well known that people who suffer from tinnitus often feel ignored by their GPs, and are dissatisfied with the service they received [26, 48]. This study found that those who felt supported by their oncologists spoke less fearfully about their ototoxic symptoms. However, the lack of information and awareness about ototoxicity may expand to clinical staff in addition to patients, and there is an opportunity for future research to investigate this.

The themes suggest that, in general, the psychosocial impact of ototoxicity has a greater impact on QoL compared to the physical symptoms for the participants in this study. Specifically, the confusion and lack of understanding about what was happening when developing ototoxic symptoms was mentioned by almost all participants. When talking about their experiences of ototoxicity, it was clear that many participants felt fear. This was in relation to both a fear of their hearing deteriorating and their tinnitus deteriorating, and how they would not be able to cope. Health related fear and anxiety has consistently been shown to have a detrimental effect on QoL [49], which can also be exacerbated by a lack of knowledge of the conditions being experienced [50–53].

Throughout the course of the interviews, participants highlighted that one of the main areas in which they had been impacted by their symptoms of ototoxicity was in social situations, making it difficult for them to communicate with their friends and family. This is cause for concern as communicating and having social support from loved ones is key for aiding an individual’s coping and adjustment to chronic illness and has been shown to be a significant coping resource in cancer patients [54, 55]. By improving the information and support offered through audiological referrals and increased awareness, interventions such as hearing aids could be used in this populations to reduce this impact on social life, thus improving patients’ abilities to cope.

It may also be useful to consider including a friend or family member in future interventions/awareness initiatives. The Developmental-Contextual Model of couples with chronic illness [56] expands on the social support perspective and puts forward a dyadic approach to coping. Couples specifically interact when dealing with stressors and their interdependence affects appraisals of illness, appraisals of stressors and ways in which they cope. Due to the impact of ototoxicity on QoL being mainly social, including the partner in the promotion of awareness of ototoxic effects of chemotherapy may be a significant help to the patient not only for awareness of potential barriers to communication, but for coping and adjusting to ototoxic symptoms if they appear.

## Strengths and limitations

There are many strengths to this study; to our knowledge, this is the first study researching in depth the specific impacts ototoxicity has on QoL. Participants were from a range of socioeconomic backgrounds, ages and years since having chemotherapy. Although many participants were local to the Nottingham area, participants were from around the UK. However, as the sample was racially homogeneous, there are likely perspectives that are not included.

Furthermore, due to not having access to the participants medical records, self-reported medical history was taken which may not be reliable, especially in cases where the participants could not recall what type of chemotherapy they received. Thus, their hearing loss could be age or noise related, not from ototoxic chemotherapy.

## Conclusion

The key themes developed from this qualitative study identify the current issues adults face when experiencing chemotherapy-induced ototoxicity. From the interviews, more awareness is needed surrounding ototoxic effects and the impact this has on QoL. Specifically, social QoL and the fear and anxiety associated with the lack of awareness must be addressed when managing ototoxic symptoms. Furthermore, the experiences with clinicians have a major role in determining whether people receive guidance and support for their symptoms. Clinical staff that do not engage, refer or offer support can have a negative impact on the QoL of their patients, compared to those that listen and offer guidance, even without a referral to audiology.

## Clinical Implications

This study identified key themes and issues surround chemotherapy-induced ototoxicity, which holds potential for future research. More support is needed for those experiencing this late effect, including increased awareness, improved clinical attitudes towards ototoxicity and referrals to audiology. Furthermore, information tools such as apps and leaflets may not be the most effective way of informing everyone about ototoxicity, and thus a more personalised approach should be considered when informing patients of side effects.
